# Biparietal remodelling and total vault remodelling in scaphocephaly—a comparative study using 3d stereophotogrammetry

**DOI:** 10.1007/s00381-023-06115-8

**Published:** 2023-08-22

**Authors:** Peter Spazzapan, Miha Verdenik, Tomaž Velnar

**Affiliations:** 1 Paediatric Neurosurgery Unit, Clinical Department of Neurosurgery, University Medical Centre Ljubljana, Ljubljana, Slovenia; 2 Faculty of Medicine, University of Ljubljana, 1000 Ljubljana, Slovenia; 3 Department of Maxillofacial and Oral Surgery, University Medical Centre Ljubljana, Ljubljana, Slovenia

**Keywords:** Scaphocephaly, 3D stereophotogrammetry, Craniosynostosis, Cranial index

## Abstract

**Purpose:**

The aim of the study was to compare the results of two surgical techniques for the treatment of isolated sagittal synostosis (ISS) by means of 3D stereophotogrammetry. One technique, the Renier’s “H” technique (RHT) comprised a biparietal expansion, the other, the total vault remodeling (TVR) included also a frontal remodeling.

**Methods:**

The two groups of operated children were compared with a third control group of normocephalic children. The 3D scanning was performed in all children between 12 and 245 months of age. On each 3D image six measurements and indices have been made, with the aim of evaluating not only length and width of the head, but also the height. The cranial index (CI) was measured in a plane parallel to the nasion-tragus plane, at the intersection with the opisthocranion.

**Results:**

Each of the three groups (RHT, TVR, control group) included 28 children. The measurements that were influenced by the correction of the frontal bossing, namely the CI and the sagittal length, were closer to normocephaly after TVR than after RHT. Lesser or no statistical difference was documented in the measurements evaluating the biparietal aspect and the height of the vertex, indicating that the biparietal expansion is effective in both procedures.

**Conclusion:**

Based on our results TVR results in a better esthetical outcome, particularly in relation to the direct surgical remodeling of the frontal bossing.

## Introduction

Craniosynostosis is a common cause of cranial deformations and is associated with premature ossification of the skull sutures. The estimated incidence is 0.4/1000 births [1]. Isolated sagittal synostosis (ISS) is the most common form, accounting for 40 to 60% of single suture synostosis [1, 2]. It results in a scaphocephalic head shape, characterized by a narrowed biparietal and bitemporal width, increased cranial length and by a variable degree of occipital and frontal bossing [3–5].

Among the clinical characteristics of ISS, frontal bossing is the one that may generate a major stigmata, since the posterior narrowing can be covered by hairs. Thus, surgical efforts should be concentrated to remodel the frontal bossing to a more anatomical and imperceptible condition [6]. Controversy persists in regard to the optimal approach to address this deformity. It has been demonstrated that the frontal bossing spontaneously corrects after a middle vault expansion, though others believe that it requires direct surgical remodeling, especially in severe cases [3].

In our center two surgical techniques have been used between 2015 and 2020 for the treatment of ISS. The first procedure comprised a biparietal expansion, the second included also a bifrontal remodeling. The aim of this study was to compare these two groups of operated children and a control group of age-matched normocephalic children. For these purposes we used the technique of 3D stereophotogrammetry, a precise and inexpensive imaging tool without radiation exposure [7, 8].

## Methods

The study was retrospective and non randomized. We compared the results of two surgical techniques for ISS within the same Institution. Medical records of a series of consecutive children undergoing surgical treatment of ISS from September 2015 to April 2020 were reviewed. Preoperative assessment included a clinical examination and an ultrasound of the cranial sutures. A routine preoperative CT was not performed. All children underwent surgery between 3 and 12 months of age and were excluded from the study if the procedure was performed after 12 months of age. Children with syndromic craniosynostosis, with involvement of two or more cranial sutures, with severe surgical complications and those with an associated hydrocephalus were also excluded.

**Fig. 1:**
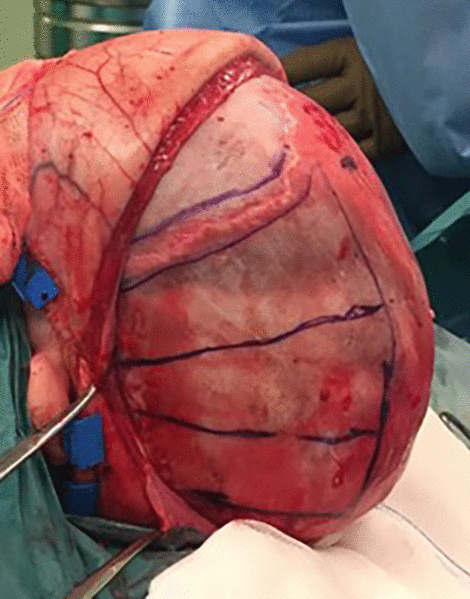
Intraoperative view of the four parietal osteotomies in a patient undergoing RHT. The coronal suture was routinely excised

All children with a diagnosis of ISS from September 2015 to March 2018 have been treated by means of Renier's “H” technique (RHT) [9]. Briefly, the synostotic suture was exposed through a posterior scalp incision. A midline flap (5 cm of width) was elevated over the sagittal sinus, which included the ossified sagittal suture. Then, four barrel stave cuts in each parietal bone were done (Fig. 1). The coronal suture was excised to prevent the possibility of a secondary coronal craniosynostosis [10]. Finally, two drains were placed and the wound was closed in layers.

**Fig. 2:**
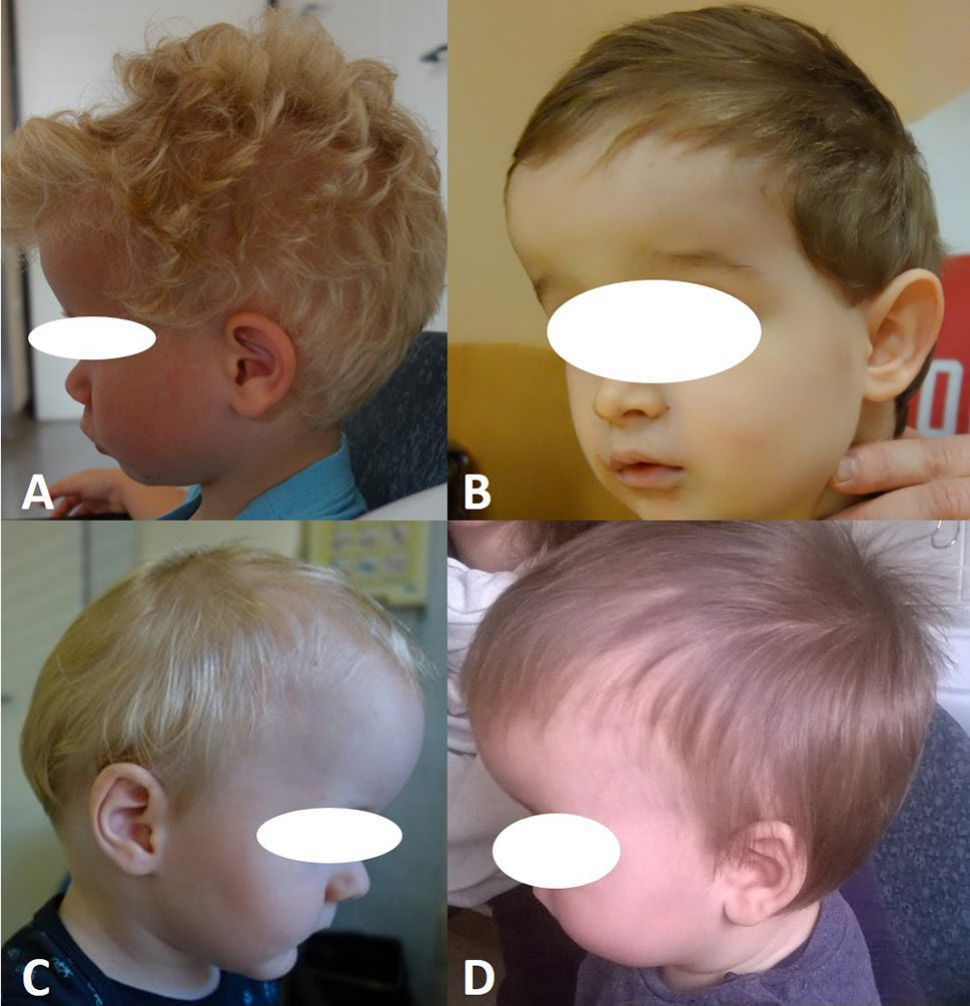
Four images showing a persisting frontal bossing at early follow-up after RHT

**Fig. 3:**
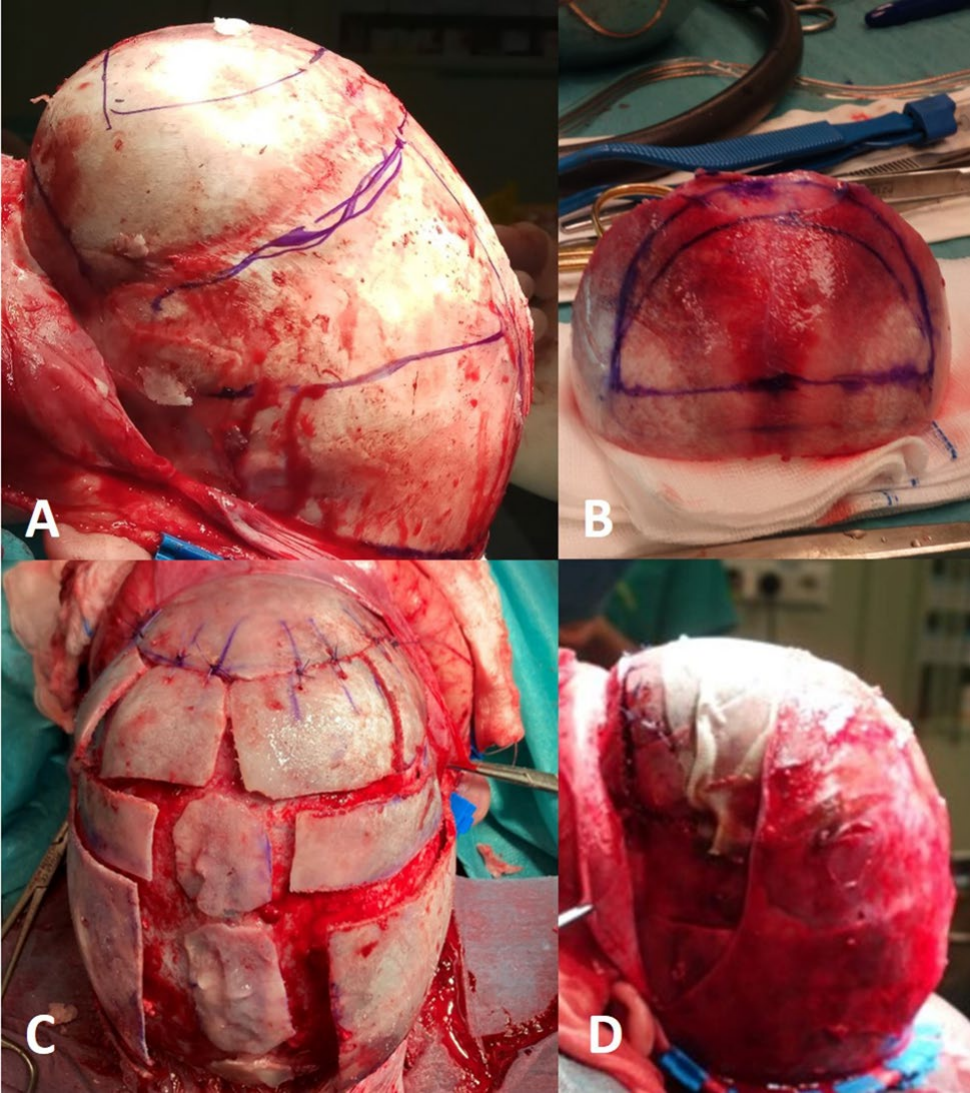
Intraoperative images of a TVR procedure. After the subperiosteal exposure of the cranial vault (**A**) the new front is designed from the bifrontal flap (**B**). The remaining pieces of bone are sutured to the new front (**C**), in order to achieve a corrected shape and a smooth surface (**D**)

At early follow-up after 3–6 months many of the operated children exhibited a persistent frontal bossing (Fig. 2), therefore the surgical protocol was modified in April 2018, introducing the procedure of total vault remodeling (TVR). In addition to the above mentioned RHT, a bifrontal craniotomy running 1 cm above the supraorbital rim was performed. The new front was designed from the bifrontal bone flap and sutured to the supraorbital rim (Fig. 3A, B). The rest of the bifrontal flap was cut in order to obtain four pieces that were sutured to the new front, thus creating a smooth surface (Fig. 3C, D).

The results of treatment were evaluated in the two groups of operated children by means of 3D stereophotogrammetry. In addition, a third control group was selected, formed by age-matched normocephalic children. These children were selected among patients visiting the outpatient clinic of other specialities at the Pediatric Clinic of the University Medical Centre Ljubljana. Normocephaly was defined based on a subjective assessment by the Authors. A child was defined as normocephalic in the absence of any clinical sign of craniosynostosis, cranial deformation or asymmetry. Before the scanning all parents signed an informed consent to participate in the study. Anonymity has been guaranteed. The 3D scanning was performed in all children between 12 and 24 months of age and at least 3 months after surgery. To avoid artifacts due to hair, children were fitted with a tight nylon cap before recording. A scanner Artec 3D Eva has been used (Artec^®^, Luxembourg) to obtain 3D images. The images in*.stl* format have been then processed in Artec Studio 16 Software^®^ (Artec^®^, Luxembourg) and analyzed with the 3dMD software (3dMD^®^, London, UK).

**Fig. 4:**
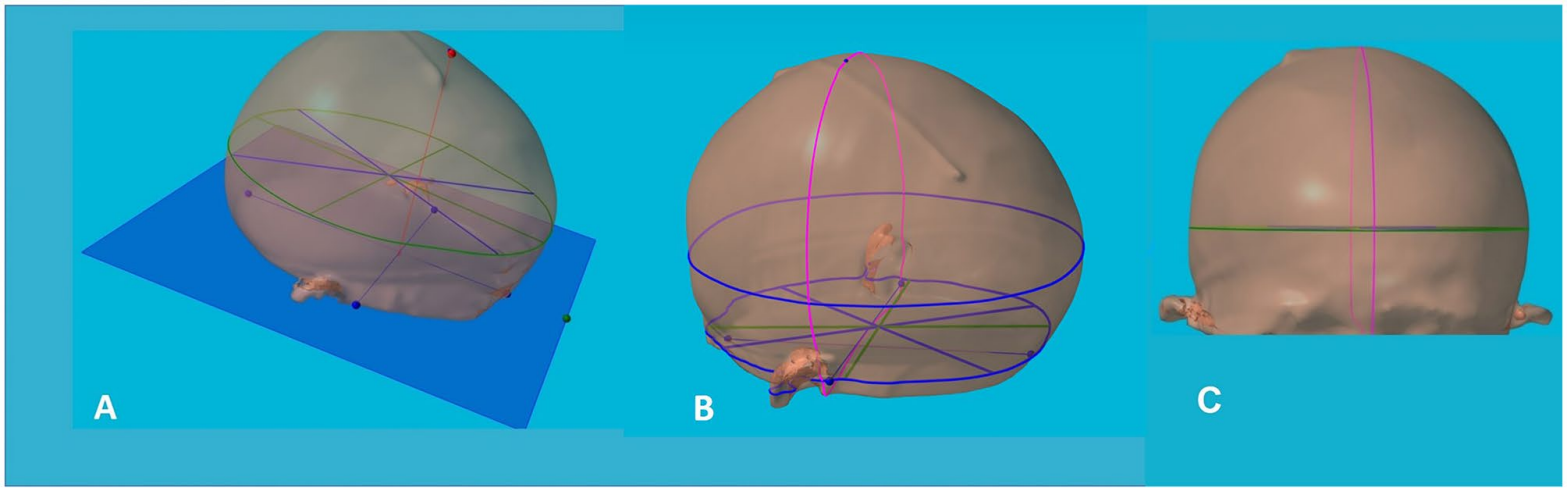
A base plane (plane-0) was designed, representing the virtual line of the skull base. It intersected the right and left tragus points and the nasion (**A**). A point on the vertex was marked (V), lying on a plane perpendicular to the plane-0 and passing through point M (*red point and red line in* A). The CI was measured in a plane parallel to the plane-0, at the intersection with the opisthocranion, the maximum posterior prominence in the occipital region (*green line in* A). The coronal length was defined as the distance form T to T, passing through the V (**B**). The sagittal length (**C**) was defined as the distance from N to the posterior midline point on plane-0

After orientating the 3D datasets in virtual space, three anatomically defined reference points have been manually selected: tragus (T), nasion (N) and the midpoint in the two tragus intersection line (M). A base plane (plane-0) representing the virtual line of the skull base, ran through the right and left tragus points and the nasion. A point on the vertex was marked (V), lying on a plane perpendicular to the plane-0, through point M (Fig. 4A).

Using these reference points, the plane-0 was shifted up to the level of the opisthocranion, the maximum posterior prominence in the occipital region. Here we measured the cranial index (CI, ratio of maximum cranial width/maximum cranial length) (Fig. 4A). According to previous studies [4, 11–13], the CI at this level captures both the frontal and occipital bossing and thus represents the maximum cranial length. Furthermore, we measured the coronal length (from T to T passing through V, Fig. 4B) and the sagittal length (from N to the midline posterior point on plane-0, Fig. 4C).

Three additional cranial indices have been calculated, which considered not only length and width of the head, but also height:

- Coronal-sagittal index (CSI): coronal circumference/sagittal circumference (expected to be lower in scaphocephalic than in normocephalic children).
- Coronal circumferential index (CCI): distance T-T/coronal circumference (expected to be lower in normocephalic than in scaphocephalic children).
- Vertex height index (VHI): distance T-T/distanceM-V (expected to be lower in normocephalic than in scaphocephalic children).

Differences in the means of the measured variables among the participants of the three groups were investigated using one-way analysis of variance (ANOVA). The results were presented as estimated means and the estimated difference between means, along with the corresponding 95% confidence interval (CI). For pairwise comparisons of the measured variables between the groups, the Hochberg test was utilized. Data were processed using the statistical software SPSS 25 (SPSS Inc., Illinois, USA). P-values less than 0.05 were considered statistically significant.

Institutional ethics approval was obtained for this research by the Medical Ethics Committee of the Republic of Slovenia (protocol n.0120–291/2021/6). Written consent was obtained for all photographs taken and for their use in publication. All subjects were enrolled upon a consent form signed by their parents, in accordance with the Helsinki Declaration of 1975, as amended in 1983.

## Results

**Fig. 5:**
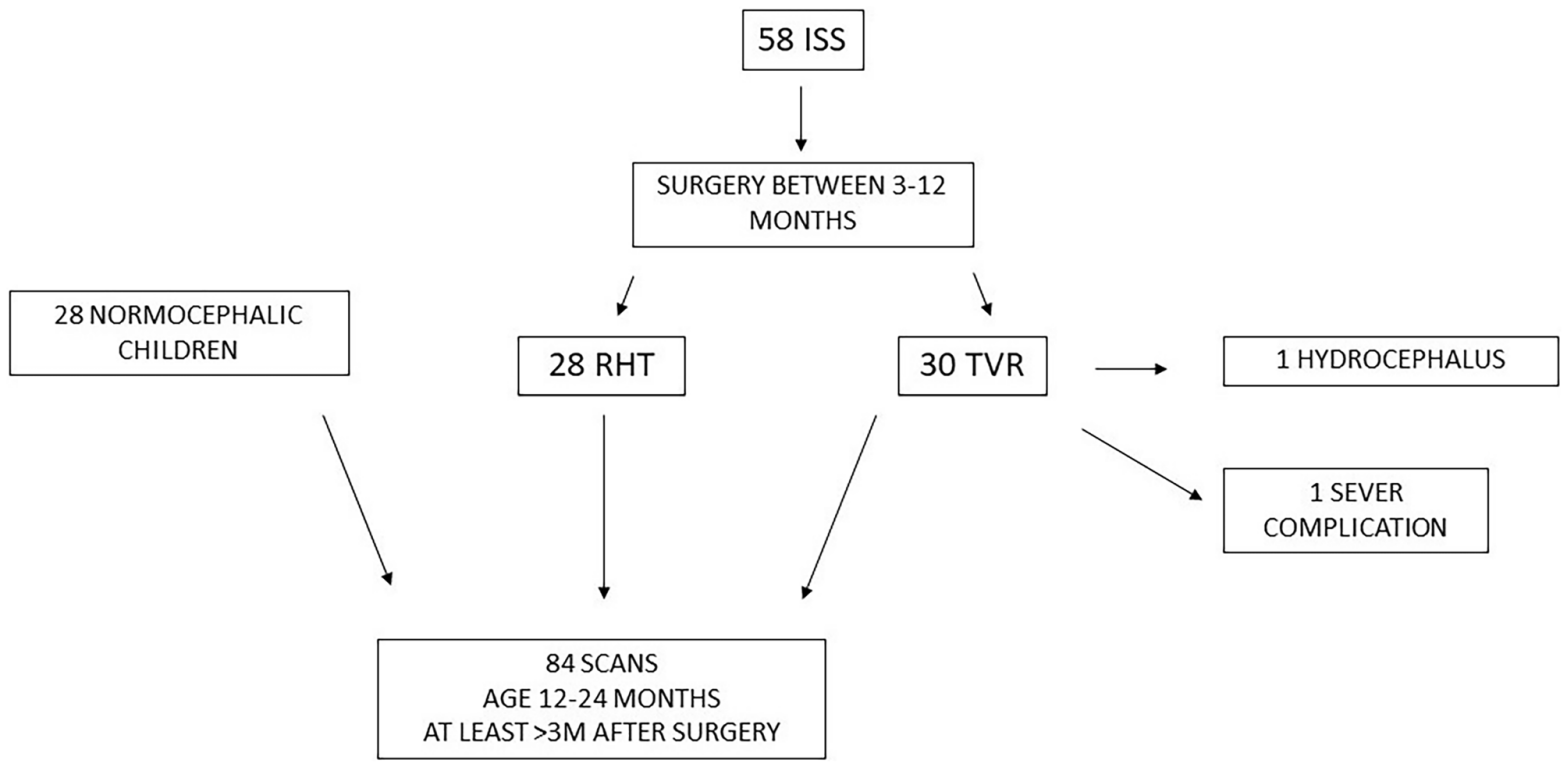
The flowchart showing how the study selection process was constructed based on the series of 58 consecutively operated cases of ISS

Overall, in the study period, 58 children were surgically treated at our Institution for ISS. The surgical protocol was modified from RHT to TVR in April 2018, when 28 children were operated by means of RHT. The enrolment of children in the TVR group and in the control group continued until the number of 28 was reached, in order to have three homogeneous groups with the same number of children. Of the 58 operated children, two were excluded from the study, one because of an associated posthaemorrhagic prematurity-related hydrocephalus, the other because of a postoperative sepsis and cellulitis of the scalp, which caused an abnormal scarring of the soft tissues. Following a strict surgical protocol, all children included in the study received a blood transfusion during surgery or in the early postoperative period. The selection and screening process is shown in the flowchart in Fig. 5.

Each of the three groups (RHT, TVR and control group) was formed by 28 children and on each 3D image six measurements have been obtained (Table 1). Median age at surgery was 5.1 months, while median age at 3D scanning was 20.2 months of age. The median age at surgery and at 3D scanning did not differ among the three groups of children.

**Table 1:** Comparisons of means of measured variables within the three groups (RHT, TVR, control group)

Measured variable/group		N	Mean value	95% Confidence interval for mean	Estimated average difference (RHT-CONTROL; TVR-CONTROL)	95% Confidence interval for estimated average difference	P value
**CI**							< 0,001
	RHT	28	0,7286	0,7152; 0,7419	-0,0530	-0,0750; -0,0309	< 0,001 (RHT vs CONTROL)
	TVR	28	0,7578	0,7444; 0,7712	-0,0237	-0,0458; -0,0017	0,033 (TVR vs CONTROL)
	CONTROL	28	0,7815	0,7658; 0,7973			
**SAGITTAL LENGHT**							0,002
	RHT	28	386,1	380,8; 391,5	14,4	4,1; 24,7	0,005 (RHT vs CONTROL)
	TVR	28	371,9	364,9; 378,8	0,1	-10,2; 10,4	1 (TVR vs CONTROL)
	CONTROL	28	371,7	364,3; 379,1			
**CORONAL LENGHT**							0,086
	RHT	28	343,6	339,4; 347,9	0,95	-9,13; 11,03	0,967 (RHT vs CONTROL)
	TVR	28	334,5	329,0; 340,0	-8,21	-18,29; 1,87	0,125 (TVR vs CONTROL)
	CONTROL	28	342,7	333,8; 351,6			
**CSI**							0,004
	RHT	28	0,8905	0,8817; 0,8992	-0,0318	-0,0529; -0,0107	0,002 (RHT vs CONTROL)
	TVR	28	0,9003	0,8877; 0,9130	-0,0219	-0,0431; -0,0008	0,041 (TVR vs CONTROL)
	CONTROL	28	0,9223	0,9044; 0,9401			
**CCI**							0,007
	RHT	28	0,3473	0,3382; 0,3564	-0,0200	-0,0357; -0,0042	0,010 (RHT vs CONTROL)
	TVR	28	0,3477	0,3401; 0,3553	-0,0196	-0,0354; -0,0039	0,012 (TVR vs CONTROL)
	CONTROL	28	0,3673	0,3543; 0,3803			
**VHI**							0,002
	RHT	28	0,8741	0,8076; 0,9406	-0,1079	-0,1751; -0,0407	0,001 (RHT vs CONTROL)
	TVR	28	0,9221	0,9073; 0,9369	-0,0599	-0,1271; 0,0073	0,087 (TVR vs CONTROL)
	CONTROL	28	0,9820	0,9507; 1,0134			

**Fig. 6:**
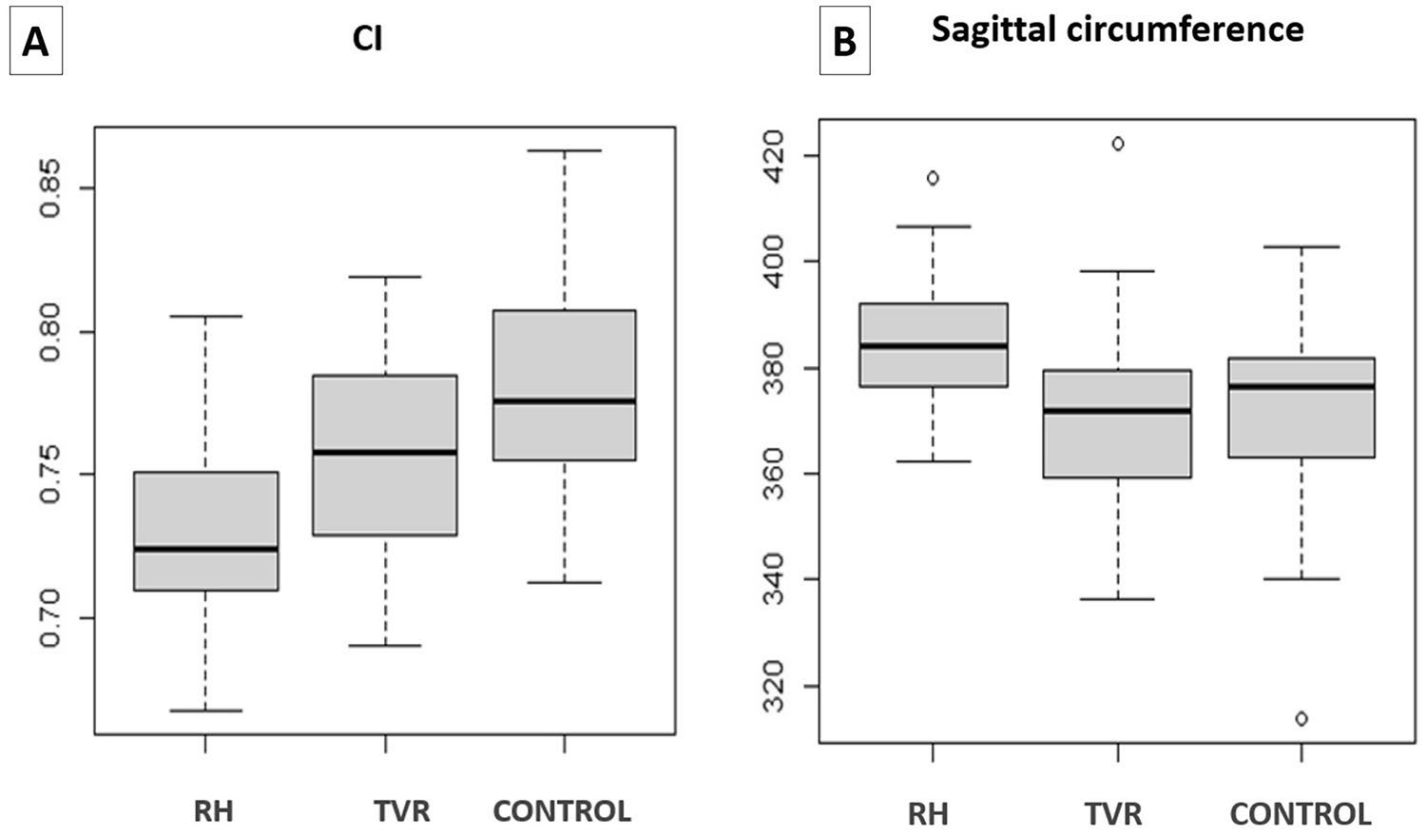
The CI and the sagittal length were the two measurements that mostly captured the prominence of the frontal bossing. The statistical difference between the TVR and the RHT groups was significant for the CI (p = 0.011) (**A**) and for the sagittal length (p = 0.008) (**B**). The values were closer to the control group after the TVR, compared to the RHT

The difference in mean values of CI measured at the level of the opisthocranion was statistically significant among the three groups (ANOVA, p < 0,001) (Fig. 6A). Children after RHT had on average 0.0530 (95% CI: 0.0309–0.0750) lower CI than children in the control group (p < 0.001). Children after TVR had on average 0.0237 (95% CI: 0.0017–0.0458) lower CI than children in the control group (p = 0.033). The difference in the mean value of the CI between the RHT and TVR groups was also statistically significant (Hochberg test, p = 0.011). Children after RHT had an average of 0.0293 (95% CI: 0.0054–0.0531) lower CI than children TVR.

The mean value of sagittal length was statistically significantly different between the three groups (ANOVA, p = 0.002) (Fig. 6B). Children operated with RHT had a mean of 14.4 mm (95% CI: 4.1–24.7 mm) higher sagittal length than children in the control group (p = 0.005). The difference in the mean value between the TVR group and the control group was not statistically significant (p = 1). The difference in the mean value of sagittal length between the RHT and TVR groups was statistically significant (Hochberg test, p = 0.008). Children after RHT had on average 14.3 mm (95% CI: 3.1–25.4) higher sagittal length than children after TVR.

**Fig. 7:**
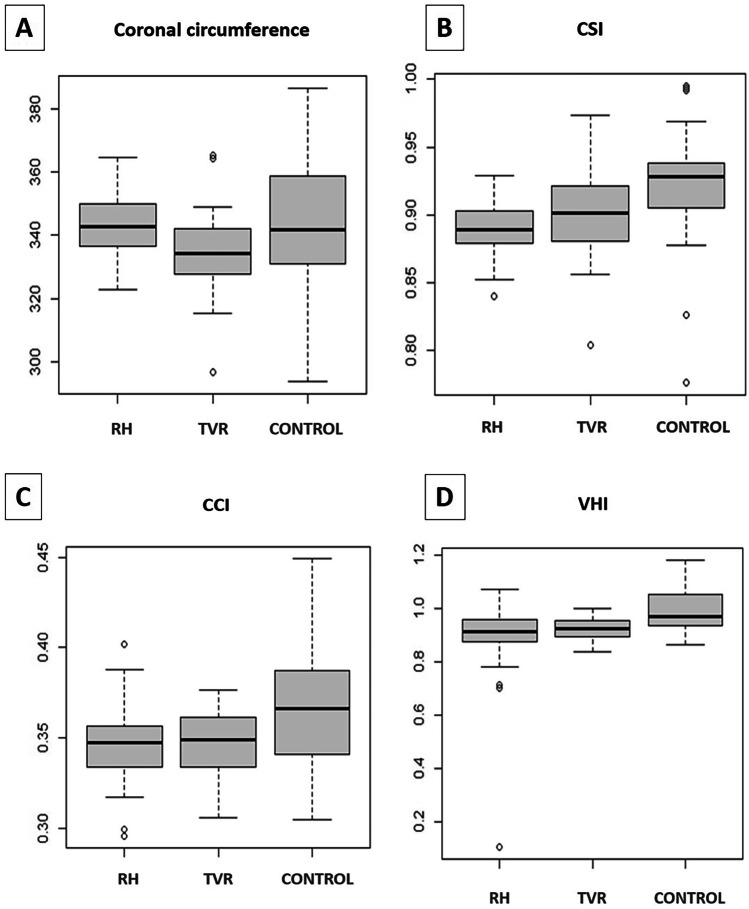
The values of the coronal length (**A**), of CSI (**B**), CCI (**C**) and VHI (**D**) did not show any significant statistical difference between the RHT and TVR groups. This demonstrated the equivalence of biparietal expansion between the two techniques. None of the two techniques was able to improve the CSI and VVI to the level of normocephalic children. CCI was lower in the two groups of operated children, compared to the control group, thus indicating an effective middle vault expansion after both procedures

The mean value of coronal length was not statistically significantly different between the three groups (ANOVA, p = 0.086) (Fig. 7A).

The difference in mean values of CSI was statistically significant among the three groups (ANOVA, p = 0.004) (Fig. 7B). Children after RHT had on average 0.0318 (95% CI: 0.0107–0.0529) lower CSI values than the control group (p = 0.002). Children after TVR had on average 0.0219 (95% CI: 0.0008–0.0431) lower CSI values than children in the control group (p = 0.041). The difference in the mean value of CSI between the RHT and TVR groups was not statistically significant (p = 0.649).

The mean value of CCI was statistically significantly different between the three groups (ANOVA, p = 0.007) (Fig. 7C). Children operated with RHT had on average 0.0200 (95% CI: 0.0042–0.0357) lower values of CCI than children in the control group (p = 0.010). Children operated by means of TVR had on average 0.0196 (95% CI: 0.0039–0.0354) lower CCI values than children in the control group (p = 0.012). The difference in the mean value of CCI between the RHT and TVR groups was not statistically significant (p = 1).

The mean value of VHI was statistically significantly different between the three groups (ANOVA, p = 0.002) (Fig. 7D). Children after RHT had a mean VHI value 0.1079 (95% CI: 0.0407–0.1751) lower than children in the control group (p = 0.001). The difference in the average values between the TVR group and the control group was not statistically significant (p = 0.087). The difference in the average VHI values between the RHT and TVR groups was not statistically significant (p = 0.297).

## Discussion

A myriad of different surgical procedures have been used for the treatment of ISS, including strip suturectomy, various methods of cranial vault remodeling, endoscopic-assisted craniectomy followed by helmet therapy and spring-assisted craniectomy [2]. We used the RHT in 28 consecutive cases of ISS between 2015 and 2018, but a slight cosmetic deformation of the frontal bone was persisting after surgery. Therefore, we had the impression that a more extensive operation with additional frontal remodeling may improve the postoperative aesthetic performance. We designed this study, to compare the craniometric results of these two groups of operated children. The results were evaluated by means of 3D stereophotogrammetry and compared with a third group of healthy, age-matched children.

Among cranial indices we used the CI, which is frequently used for reporting cosmetic results after surgical correction [14]. Since several variations exist in the methodology used to determine the CI [11, 15, 16], we adopted the method validated by Ruiz-Correa [3], that calculates the CI in a plane parallel to the plane-0, at the level of the opisthocranion. This plane captures both the frontal bossing and the occipital bossing [5] and thus provides improved sensitivity and specificity for the scaphocephalic deformation.

Since CI remains significantly limited to fully address the cranial shape [14–18], we also measured the sagittal length, the coronal length and three cranial indices: CSI, CCI and VHI. CSI evaluates the relation between the biparietal width and the sagittal elongation of the head. This index is lower in ISS compared to normocephalic children. CCI evaluates the severity of biparietal narrowing in a coronal plane, while VVI evaluates the cranial height and the amount of the vertex flattening, which is typical for ISS: both are higher in ISS compared to normocephalic children:

These three indices and the coronal length did not show statistically significant differences between the RHT and TVR groups. This fact demonstrates that biparietal expansion is equally achieved in both procedures. Despite this, when comparing separately the RHT and TVR groups with the control group, none of the two techniques improved CSI and VVI to the level of normocephalic children. This is not true for the CCI, which was lower in the two groups of operated children compared to the normocephalic group, thus indicating that both techniques were able to guarantee a clear improvement in terms of biparietal expansion.

On the other hand, the two measurements that addressed the frontal bossing were significantly different between the RHT and TVR groups. The statistical difference between the TVR and the RHT group was significant for CI (p = 0.011) and sagittal length (p = 0.008) and the values in the TVR group were closer to those of the group of normocephalic group.

We must underline that in comparison to the previously described metrics [20–22], none of the indices used in this study directly assessed the severity of the frontal bossing. In fact, our aim was to define the whole cranial vault shape and to determine the different aesthetical outcomes between RHT and TVR. In this sense, indeed the frontal bossing represents an important parameter, and its severity is captured by the CI measured with the method we have utilized [4].

The statistical power of the indices used in the study was not analysed with a ROC curve, since these indices were not validated through a preoperative comparison of values across control and disease groups. Some uncertainty remains indeed, whether these metrics are trending the disease state, the surgical interventions, or some other variable. Despite these limitations, we can state that, when differentiated along these indices and at the studied time point, the values between the control group and the two groups of operated children appear significantly different. An image-based validation of the measurements used in the study is presented in Table 2. Our results are in line with a review of the Literature [14], which compared the results of four different surgical techniques for ISS and demonstrated that total cranial vault remodeling and endoscopic-assisted craniectomy followed by helmet therapy showed the best aesthetic outcome in terms of improved CI. Similar results have been outlined by a systematic review [19], which demonstrated that aesthetic outcome in terms of improved CI appears to be better in patients who undergo remodeling of the frontal bossing, compared to less invasive procedures.

**Table 2:**
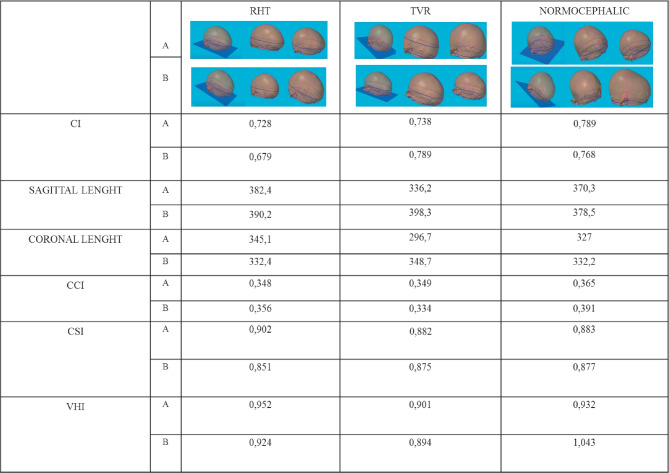
Image-based validation of the six indices used in the study. Six representative 3D scans, two (A and B) for each group (RHT, TVR, control group) are presented. The differences can be visually appreciated and are confirmed by the different values of CI, sagittal length, coronal length, CSI, CCI and VHI. The values in lines A and B refer respectively to the scans presented in line A and B. The values of CI and Sagittal length were significantly different among the three groups (ANOVA) and between the two groups of operated children, thus confirming the influence of the frontal remodeling on both measurments

Despite these results, a consensus regarding the optimal treatment of ISS is far from being achieved. In particular, regarding the issue of frontal bossing many questions remain unresolved. Yen compared two groups of operated children, one with frontal remodeling and one without it, with a third control group [20]. One year after surgery both groups had worse CI compared to controls, though the group of frontal remodeling showed better frontal bossing measures. At two years of age, patients with frontal remodeling normalized frontal morphology to a greater extent than those without frontal remodeling, despite neither of the two groups achieved complete normalization.

To counterbalance these results, several studies have observed that frontal bossing spontaneously corrects following a middle vault expansion [17, 21, 23, 24]. In this way, the possible complications of a frontal craniotomy are avoided. Proponents believe that after the release of the ISS, the cranial vault expands in both parietal directions and the compensatory growth in the frontal region decreases [21, 23, 24]. This long-term spontaneous improvement has been documented with 3D images [21]: there was no significant difference with age-comparable controls two years after the middle vault expansion.

We adopted TVR after observing a persisting frontal bossing at early follow-up in RHT patients. Our early results were satisfying and avoided the uncertain and doubtful waiting time to natural correction of the frontal bossing.

One drawback of our study is that our measurements have all been performed early in the postoperative period and did not assess the long-term outcomes. Despite this, careful analysis of craniometric data, including CI, showed that 18 months appears to be the turning point after which the CI stabilizes and remains unchanged during subsequent years [1]. In this sense, we can interpret our results as very close to what is expected to be the long term outcome.

A common criticism to the remodeling of the whole cranial vault is the invasiveness of the procedure and the increased risk of complications. In literature there is a clear tendency toward less invasive techniques [26] and endoscopic suturectomy followed by helmet therapy appears significantly less morbid compared to extensive calvarial remodeling procedures [1, 16, 18, 25, 26]. Nevertheless, endoscopy is far from being a gold standard in treatment of ISS and open surgery offers many advantages, such as early excellent cosmetic results without the need for postoperative helmet therapy.

In our series the major invasiveness of TVR compared to RHT did not result in a larger number of surgical complications. The rate of blood transfusions between the two groups did not differ. Thus, we would recommend taking in consideration the possibility of a surgical remodeling of the frontal bone in all ISS, or at least in those presenting with a severe bossing deformity.

In our study all measurements have been done using 3D stereophotogrammetry, which is useful for describing craniofacial dimensions, shapes and evaluating treatment outcomes [7, 27]. These are all major advantages compared to more simplistic analyses performed with direct anthropometry. Previous studies have shown a strong correlation and negligible errors between measurements made by 3D photographs and CT scans [28], thus making 3D imaging a reliable, fast and precise method to quantify cranial dimensions [27, 29]. It represents a valid alternative to CT, which requires sedation and is associated with radiation exposure to the developing brain [7, 30].

## Conclusions

Despite no consensus exists regarding the ideal surgical strategy for the treatment of ISS, our results show that CI and sagittal length are closer to normocephalic values after TVR, when compared to RHT. The values of CSI, CCI and VHI are not statistically different between TVR and RHT, proving that the biparietal expansion is equally achieved by both techniques. Since the frontal bossing represents an important and visible deformity, all efforts should be directed toward its early correction and TVR represents an effective method to achieve this result.

## Data Availability

The datasets generated during and/or analyzed during the current study are available from the corresponding author on reasonable request.

## References

[1] Jimenez DF, Barone CM (2012). Endoscopic technique for sagittal synostosis. Childs Nerv Syst.

[2] Di Rocco F, Gleizal A, Szathmari A, Beuriat PA, Paulus C, Mottolese C (2019). Sagittal suture craniosynostosis or craniosynostoses? The heterogeneity of the most common premature fusion of the cranial sutures. Neurochirurgie.

[3] Wagner W, Wiewrodt D (2008). A simple technique for the correction of frontal bossing in synostotic scaphocephaly. Childs Nerv Syst.

[4] Ruiz-Correa S, Sze RW, Starr JR, Lin HT, Speltz ML, Cunningham ML, Hing AV (2006). New scaphocephaly severity indices of sagittal craniosynostosis: a comparative study with cranial index quantifications. Cleft Palate Craniofac J.

[5] Isaac KV, Meara JG, Proctor MR (2018). Analysis of clinical outcomes for treatment of sagittal craniosynostosis: a comparison of endoscopic suturectomy and cranial vault remodeling. J Neurosurg Pediatr.

[6] Raposo-Amaral CE, Denadai R, Takata JP, Ghizoni E, Buzzo CL, Raposo-Amaral CA (2016). Progressive frontal morphology changes during the first year of a modified Pi procedure for scaphocephaly. Childs Nerv Syst.

[7] Linz C, Meyer-Marcotty P, Böhm H, Müller-Richter U, Jager B, Hartmann S, Reichert C, Kochel J, Schweitzer T (2014). 3D stereophotogrammetric analysis of operative effects after broad median craniectomy in premature sagittal craniosynostosis. Childs Nerv Syst.

[8] Wong JY, Oh AK, Ohta E, Hunt AT, Rogers GF, Mulliken JB, Deutsch CK (2008). Validity and reliability of craniofacial anthropometric measurement of 3D digital photogrammetric images. Cleft Palate Craniofac J.

[9] Di Rocco F, Knoll BI, Arnaud E, Blanot S, Meyer P, Cuttarree H, Sainte-Rose C, Marchac D (2012). Scaphocephaly correction with retrocoronal and prelambdoid craniotomies (Renier's "H" technique). Childs Nerv Syst.

[10] Arnaud E, Capon-Degardin N, Michienzi J, Di Rocco F, Renier D (2009). Scaphocephaly: part II: secondary coronal synostosis after scaphocephalic surgical correction. J Craniofac Surg.

[11] Albright AL, Towbin RB, Schultz BL (1996). Long term outcome after sagittal synostosis operations. Pediatr Neurosurg.

[12] Friede H, Lauritzen C, Figueroa AA (1996). Roentgen cephalometric follow up after early osteotomies in patients with scaphocephaly. J Craniofac Surg.

[13] Guimaraes-Ferreira J, Gewalli F, David L, Olsson R, Friede H, Lauritzen CGK (2001). Clinical outcome of the modified pi-plasty procedure for sagittal synostosis. J Craniofac Surg.

[14] Bonfield CM, Lee PS, Adamo MA, Pollack IF (2014). Surgical treatment of sagittal synostosis by extended strip craniectomy: cranial index, nasofrontal angle, reoperation rate, and a review of the literature. J Craniomaxillofac Surg.

[15] Dvoracek LA, Skolnick GB, Nguyen DC, Naidoo SD, Smyth MD, Woo AS, Patel KB (2015). Comparison of Traditional versus Normative Cephalic Index in Patients with Sagittal Synostosis: Measure of Scaphocephaly and Postoperative Outcome. Plast Reconstr Surg.

[16] Shah MN, Kane AA, Petersen JD, Woo AS, Naidoo SD, Smyth MD (2011) Endoscopically assisted versus open repair of sagittal craniosynostosis: the St. Louis Children's Hospital experience. J Neurosurg Pediatr 8(2):165–70. 10.3171/2011.5.PEDS1128 10.3171/2011.5.PEDS1128 21806358

[17] Marsh JL, Jenny A, Galic M, Picker S, Vannier MW (1991) Surgical management of sagittal synostosis. A quantitative evaluation of two techniques. Neurosurg Clin N Am 2(3):629–640. PMID: 1821309 1821309

[18] Thomas GP, Johnson D, Byren JC, Jayamohan J, Magdum SA, Richards PG, Wall SA (2015). Long-term morphological outcomes in nonsyndromic sagittal craniosynostosis: a comparison of 2 techniques. J Craniofac Surg.

[19] Thwin M, Schultz TJ, Anderson PJ (2015). Morphological, functional and neurological outcomes of craniectomy versus cranial vault remodeling for isolated nonsyndromic synostosis of the sagittal suture: a systematic review. JBI Database System Rev Implement Rep.

[20] Yen DW, Nguyen DC, Skolnick GB, Naidoo S, Smyth MD, Patel KB, Woo AS (2019). Evaluation of Direct Surgical Remodeling of Frontal Bossing in Patients With Sagittal Synostosis. J Craniofac Surg.

[21] Khechoyan D, Schook C, Birgfeld CB, Khosla RK, Saltzman B, Teng CC, Ettinger R, Gruss JS, Ellenbogen R, Hopper RA (2012). Changes in frontal morphology after single-stage open posterior-middle vault expansion for sagittal craniosynostosis. Plast Reconstr Surg.

[22] Harrison LM, Ferrari EJ, Mathew DP, Derderian CA, Hallac RR (2023). Three-dimensional Analysis of Facial Asymmetry in Unilateral Lambdoid Craniosynostosis. Cleft Palate Craniofac J.

[23] Amm CA, Denny AD (2005). Correction of sagittal synostosis using foreshortening and lateral expansion of the cranium activated by gravity: surgical technique and postoperative evolution. Plast Reconstr Surg.

[24] Panchal J, Marsh JL, Park TS, Kaufman B, Pilgram T, Huang SH (1999). Sagittal craniosynostosis outcome assessment for two methods and timings of intervention. Plast Reconstr Surg.

[25] Hinojosa J, Esparza J, Muñoz MJ (2007). Endoscopic-assisted osteotomies for the treatment of craniosynostosis. Childs Nerv Syst.

[26] Schulz M, Liebe-Püschel L, Seelbach K, Paulikat L, Fehlhaber F, Schwarz K, Blecher C, Thomale UW (2021). Quantitative and qualitative comparison of morphometric outcomes after endoscopic and conventional correction of sagittal and metopic craniosynostosis versus control groups. Neurosurg Focus.

[27] Brons S, van Beusichem ME, Bronkhorst EM, Draaisma JM, Bergé SJ, Schols JG, Kuijpers-Jagtman AM (2014) Methods to quantify soft tissue-based cranial growth and treatment outcomes in children: a systematic review. PLoS One 27;9(2):e89602. 10.1371/journal.pone.0089602 10.1371/journal.pone.0089602 PMC3937373 24586904

[28] McKay DR, Davidge KM, Williams SK, Ellis LA, Chong DK, Teixeira RP, Greensmith AL, Holmes AD (2010). Measuring cranial vault volume with three-dimensional photography: a method of measurement comparable to the gold standard. J Craniofac Surg.

[29] Schaaf H, Pons-Kuehnemann J, Malik CY, Streckbein P, Preuss M, Howaldt HP, Wilbrand JF (2010). Accuracy of three-dimensional photogrammetric images in non-synostotic cranial deformities. Neuropediatrics.

[30] Agrawal D, Steinbok P, Cochrane DD (2006). Diagnosis of isolated sagittal synostosis: are radiographic studies necessary?. Childs Nerv Syst.

